# Production of renewable long-chained cycloalkanes from biomass-derived furfurals and cyclic ketones†

**DOI:** 10.1039/c8ra01723a

**Published:** 2018-04-12

**Authors:** Qiying Liu, Caihong Zhang, Ning Shi, Xinghua Zhang, Chenguang Wang, Longlong Ma

**Affiliations:** CAS Key Laboratory of Renewable Energy, Guangzhou Institute of Energy Conversion, Chinese Academy of Sciences Guangzhou 510650 P. R. China liuqy@ms.giec.ac.cn zhangxh@ms.giec.ac.cn +86-20-87057737 +86-20-87057751 +86-20-87057790; University of Chinese Academy of Sciences Beijing 100049 P. R. China

## Abstract

Developing renewable long-chain cycloalkanes from lignocellulosic biomass is of significance because it offers huge resource storage, wide applications in aviation/diesel fuels and mitigation of CO_2_ emissions. In this paper, cycloalkanes with carbon chain lengths of 13–18 were produced from biomass-derived furfural species (furfural and 5-hydroxymethylfurfural) and cyclic ketones (cyclopentanone and cyclohexanone) *via* aldol condensation, followed by hydrogenation to saturate the C=C and C=O bonds, and hydrodeoxygenation to remove oxygen atoms. The aldol condensation of the furfural species with cyclic ketones was catalyzed by NaOH and the target condensation intermediates were obtained in yields of more than 90% at room temperature (30 °C) with a short reaction time (40 min). By using amorphous zirconium phosphate combined with Pd/C as the catalyst, liquid cycloalkanes were produced at the optimal conditions with a yield of 76%. When the combined solid catalyst was reused, the target products reduced after the second run but the initial yield could be largely recovered by recalcination of the spent zirconium phosphate. Considering that cyclopentanone and cyclohexanone can be easily produced from furfural (originating from hemicellulose) and phenol (originating from lignin), respectively, this condensation has the potential to achieve the integrated conversion of biomass-derived cellulose, hemicellulose and lignin to jet fuel and/or diesel additives.

## Abstract 2

Renewable cyclic alkanes were produced in high yield *via* condensation of biomass-based furfurals and cyclic ketones followed by hydrodeoxygenation.

## Graphical abstract

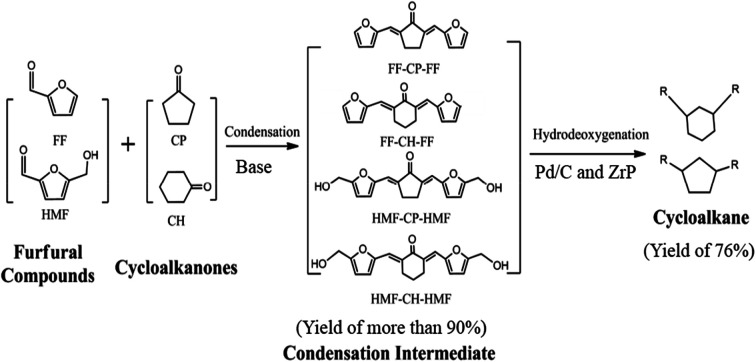

## 1. Introduction

Lignocellulosic biomass is a renewable resource with huge storage, wide distribution and fast carbon cycles during biomass growth and utilization. Biomass-derived fuels and chemicals are regarded as an alternative to fossil energy, and their development has obvious significance for environmental protection and reduced dependence on fossil energy. Therefore, converting lignocellulosic biomass into fuels has attracted worldwide attention during recent decades.^1–4^

Cellulose, hemicellulose and lignin are the three main components of a typical lignocellulosic biomass, however, the monosaccharide units (typically, glucose and xylose) and derivatives (5-hydroxymethylfurfural (HMF) and furfural (FF)) are limited to carbon chains of length 6, while the phenolic units (typically, phenol-, guaiacol- and syringyl-based primary structures) are limited to carbon chains of length 9. This results in the fuels from direct hydrodeoxygenation of these platforms having short carbon chains.^5,6^ To synthesize long-chained alkanes with carbon numbers of more than 10, for jet fuel and diesel use, condensations between suitable biomass-derived platforms with special groups are necessary to extend the carbon chains.^6–9^ Typically, the production of long-chained alkanes involves three steps: depolymerization of the lignocellulosic biomass into oxygenated platforms (FF, HMF, levulinic acid, γ-valerolactone and 2-methylfuran *et al.*); fuel precursor formation by condensation; and hydrodeoxygenation of the fuel precursors.^9,10^ Herein, the second step of platform condensation is the key to obtain structurally diversified fuel precursors; the desired platforms and condensation methods must be chosen in order to produce versatile hydrocarbon fuels by hydrodeoxygenation.^11^ Aldol condensations of FF/HMF and/or their derivatives with carbonyl groups like acetone,^12–16^ hydroxyacetone,^17^ butaldehyde,^18^ 2-butanone,^19^ methyl isobutyl ketone,^20^ levulinic acid^11,21,22^ and angelica lactone^23^ have commonly been conducted to obtain the C_9_–C_16_ fuel precursors, but these carbonyl compounds originate from fossil resources or are produced from biomass by multi-step reactions, leading to low efficiency and high cost for the entire synthesis. As an alternative, hydroxyalkylation-alkylation of FF and 2-methylfuran obtained by hydrodeoxygenation of FF was reported to produce C_15_ fuel precursors with a branched carbon framework.^24–27^ Despite the fact that the precursor could be converted into branched long-chain hydrocarbons, the main drawback was that only hemicellulose (the least abundant component in a typical lignocellulosic biomass) could be utilized. Interestingly, alternative routes including direct C–C bond formation by condensation of monosaccharides/furfurals,^28^ α-alkylation of ketones with 1-butanol,^29^ self-coupling by two HMF molecules^30^ and oligomerization of angelica lactone^31,32^ were used to produce C_10_–C_15_ precursors with high yields, and hydrodeoxygenation to the relative long-chained hydrocarbons was then conducted over supported metal catalysts. Instead of the common furfurals, these routes extended the platform molecules for target hydrocarbons by using diversified structures.

Compared to branched and normal hydrocarbons with long chains, cyclic counterparts show particular advantages in view of their low freezing points and high energy densities, which are preferential for drop-in jet and/or diesel fuels.^33^ Carbonyl-containing HMF and FF are the two main platforms which can be easily produced from biomass through a hydrolysis–dehydration process. On the other hand, cyclopentanone (CP) and cyclohexanone (CH) are two important cyclic ketones that have special carbons with α-Hs. In particular, CP could be selectively produced by hydrogenation-rearrangement of hemicellulose-derived FF,^34,35^ and CH could be prepared with very high yield from lignin-derived phenol.^36,37^ Thus, production of cyclic hydrocarbons with long chains from these furfural species and cyclic ketones are attractive for the integrated utilization of biomass.^38–40^

**Scheme 1:**
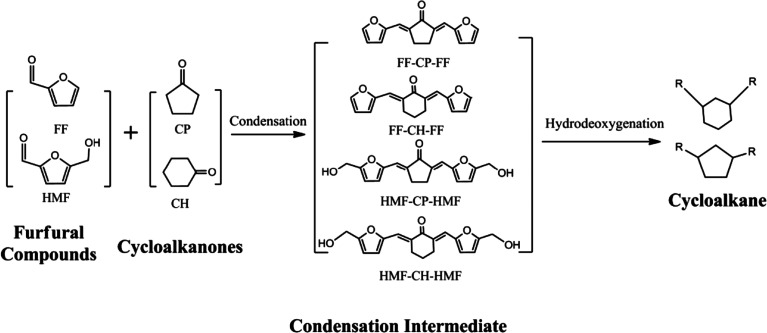
Routes for long-chained cycloalkane production from furfural species and cyclic ketones.

Here, we developed a new route for the production of cycloalkanes with carbon chain lengths of 13–18 from FF/HMF and CP/CH (Scheme 1). Aldol condensations of these furfural species with cyclic ketones (CP and CH) were first conducted over a base catalyst to produce the oxygenated precursors with cyclic structures. Then these precursors were hydrogenated to thermally stable intermediates by saturating the C=C and C=O bonds over a metal catalyst. Finally they were transformed to the long-chained cycloalkanes by using a catalyst of metal combined with solid acid. The reaction parameters of each step were screened and the pathway for long-chained cycloalkane formation is discussed.

## 2. Experimental section

### 2.1. Materials

FF (99%), HMF (98%), CP (99%), CH (99%), ZrOCl_2_·8H_2_O, Pt/C (5 wt%), Pd/C (5 wt%) and Ru/C (5 wt%) were purchased from Shanghai Crystal Pure Reagent Co., Ltd., China. Analytical grade NaOH, KOH, Ba(OH)_2_, LiOH, K_2_CO_3_, Na_2_CO_3_, Mg(OH)_2_, H_3_PO_4_ and NH_4_H_2_PO_4_ were purchased from Tianjin Fuchen Chemical Reagent Factory, China. Nb_2_O_5_·H_2_O was purchased from the Conghua Tantalum Niobium Factory (Guangzhou, China). All these chemicals and reagents were used without further purification.

### 2.2. Catalyst preparation

#### Preparation of niobium phosphate (NbP)

6 g Nb_2_O_5_·H_2_O was added into 100 mL 5.0 mol L^−1^ H_3_PO_4_ aqueous solution and vigorously stirred for 48 h at room temperature. Then the solid residue was collected by filtration and fully washed with deionized water until pH = 5. This was followed by drying at 120 °C and calcination at 400 °C for 3 h (heating ramp of 5 °C min^−1^) in static air before use.

#### Preparation of zirconium phosphate (ZrP)

10 g ZrOCl_2_·8H_2_O and 7.14 g NH_4_H_2_PO_4_ were each dissolved in 100 mL deionized H_2_O to obtain two transparent solutions. The two solutions were mixed at the calculated molar ratio of P/Zr = 2 and reacted for 8 h at room temperature under agitation. The white precipitate was filtered, fully washed with deionized water, dried at 100 °C overnight, and calcined at a temperature of 400 °C for 4 h (heating ramp of 5 °C min^−1^) in static air before use.

### 2.3. Catalyst characterization

X-ray powder diffraction (XRD) patterns of catalysts were obtained using an X-ray diffractometer (X'Pert Pro MPD, Philip) with Cu Kα radiation (*λ* = 0.154 nm) operated at 40 kV and 100 mA.

The Brunauer–Emmett–Teller (BET) specific surface areas were obtained from N_2_ isothermal adsorption–desorption measurements at 77 K using a QUADRASORB SI-MP-10/PoreMaster analyzer equipped with QuadraWin software. The mesoporous volumes and pore size distributions were calculated by the desorption branch using the Barrett–Joyner–Halenda (BJH) method.

Ammonia-temperature programmed desorption (NH_3_-TPD) was conducted in a U-tube quartz reactor (inner diameter 0.6 cm) using an ASIQACIV200-2 automatic physical/chemical adsorption analyzer (Quantachrome, US). Catalyst (0.15 g) was loaded into the U-tube quartz reactor and heated to 400 °C for 0.5 h (heating ramp of 10 °C min^−1^) under 30 mL min^−1^ of He flow. The system was cooled to 50 °C, NH_3_ was adsorbed onto the catalyst for 0.5 h until the saturation state was obtained and then the catalyst was flushed with a He flow of 30 mL min^−1^ to remove the physically adsorbed NH_3_. The adsorption and removal processes were monitored by the TCD signals. Then the system was heated to 825 °C with a heating ramp of 10 °C min^−1^ under the same He flow. The online MS (AMETEK DYCOR LC-D200, US) was used to determine the effluent NH_3_ desorbed from the catalyst. The quantitative analysis of acidic sites was performed using a calibration loop of 250 μL in which 5% NH_3_-95% Ar mixed gas was used for the single point calibration.

Transmission electron microscopy (TEM) images were gained on a Gatan Ultra scan camera using a JEOL JEM-2100F instrument operated at 200 kV. The samples were ultrasonically dispersed in ethanol, and drops of each suspension were placed on carbon-coated copper grids and then dried in air.

Scanning electron microscopy (SEM) images were recorded using an S-4800 instrument operated at 2 kV. The samples were placed on a conductive carbon tape adhered to an aluminium sample holder.

Fourier transform infrared (FT-IR) spectra were recorded on a Bruker TENSOR27 spectrometer with a resolution of 4 cm^−1^. The samples were pelleted with KBr before measurement.

The Pd dispersion in Pd/C was tested by H_2_-chemisorption using a Quantachrome-ASIQACIV200-2 automated gas sorption analyzer. The catalyst (0.2 g) was treated at 300 °C with a heating ramp of 5 °C min^−1^ under a hydrogen atmosphere (30 mL min^−1^). To clean the catalyst surface, the catalyst was further heated to 350 °C for 0.5 h under nitrogen (30 mL min^−1^), and then cooled to 50 °C for the H_2_-chemisorption test.

### 2.4. Aldol condensation of furfural species with cyclic ketones

Aldol condensation of furfural species and cyclic ketones was conducted in a 250 mL conical flask under atmospheric pressure. Using FF and CP as example, 2 mmol FF and 1 mmol CP were introduced into the flask, followed by the addition of 15 mL H_2_O and a certain amount of basic catalyst. The condensation was conducted at 30 °C for 40–120 min in a water bath with vigorous stirring (800 rpm). After the reaction, the solid product was filtrated, fully washed with a 5% aqueous ethanol solution and dried in a freeze dryer for 48 h. A yellowish solid was finally obtained and was labeled as FF-CP-FF. The condensations of FF and CH, HMF and CP and HMF and CH were carried out by the procedure mentioned above, and the solid intermediates were labeled as FF-CH-FF, HMF-CP-HMF and HMF-CH-HMF, respectively.

The structures of the aldol condensation intermediates were identified using ^13^C NMR analysis (Bruker BioSpin GmbH) using CDCl_3_ or DMSO as the solvent.

The yield of each condensation intermediate was calculated by the following equation:1
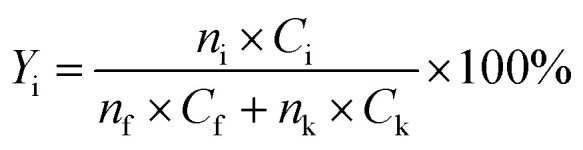
where, *Y*_i_ is the yield of condensation intermediate collected; *n*_i_, *n*_f_ and *n*_k_ represent the moles of solid intermediate i, furan and ketone introduced, respectively; and *C*_i_, *C*_f_ and *C*_k_ indicate the carbon numbers of solid intermediate i, introduced furan and ketone, respectively.

### 2.5. Hydrodeoxygenation of condensation intermediates to cycloalkanes

The hydrodeoxygenation of the condensation intermediates was carried out in a 50 mL stainless steel reactor with a mechanical stirrer. The condensation intermediate (1 mmol), 15 mL H_2_O and a certain amount of catalyst (solid acid and metal) were added to the reactor. After purging five times with H_2_, the reaction system was pressured to 4 MPa with H_2_ (room temperature). Then the reactor was heated to the target temperature and maintained at that temperature for the set time under vigorous stirring (800 rpm). After the reaction, the reactor was cooled to room temperature, and 15 mL ethyl acetate was used to extract the organic products three times. The organic layer was analyzed by gas chromatography (GC, GC-2014C, Shimadzu, Japan) and gas chromatography-mass spectrometry (GC-MS, Agilent 7890, US). For calculating the conversion percentage, the solid residue was collected by filtration, further rinsed with deionized water, collected by filtration again and dried in a freeze dryer for 48 h.

For reuse, the combined catalyst (ZrP and Pd/C) was recovered by centrifugation from the reaction mixture, thoroughly rinsed with deionized water and directly used for the next cycle. Considering that the components of the combined catalyst were hard to separate from each other, to ascertain which catalyst led to deactivation, comparative experiments were conducted by hydrothermal treatment of ZrP and Pd/C at 300 °C, followed by either recalcination at 400 °C in static air (for ZrP) or 300 °C in 30 mL min^−1^ of H_2_ flow (for Pd/C); heating ramps of 10 °C min^−1^ were used for both. For monitoring the saturation of C=C and C=O bonds by hydrogenation in the solid intermediates, the sole Pd/C catalyst was used at relatively low temperatures according to the hydrodeoxygenation process. The structures of these hydrogenation intermediates were identified using ^13^C NMR analysis (Bruker BioSpin GmbH) using CDCl_3_ or DMSO as the solvent.

The quantitative analysis of the extracted liquid products was determined by GC (SHIMADZU 2014C) with a flame ionization detector (FID) and a HP-INNOWAX column (30 m × 0.25 mm × 0.25 μm). The oven temperature was programmed initially at 60 °C for 2 min, and then ramped up to 260 °C at a heating rate of 10 °C min^−1^ and held at this temperature for another 10 min. The injector was kept at 280 °C and used in splitting mode (5 : 1) with helium as the carrier gas. Tridecane was used as the internal standard. The qualitative analysis of liquid products was carried out by GC-MS (Agilent 7890, US) used with the same capillary column and analytical conditions to those of the GC analysis. The gas products (CH_4_, C_2_H_6_ and C_3_H_8_, *etc.*) were collected with a sampling bag and analyzed by a GC 9800 chromatography tester equipped with a FID and a packed column (Porapak-Q column, 3 m × 3 mm). The quantification of gas products was conducted using a typical external standard.

The conversion of intermediates and yield of liquid product was calculated by the following equations:

2 Conversion (%) = *m*_intermediate residue_/*m*_intermediate loaded_ × 100%

3 *m*_intermediate residue_ = *m*_solid residue_ − *m*_catalyst_

4
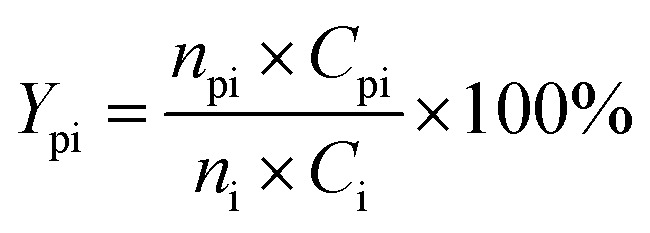
where, *m*_intermediate residue_ and *m*_intermediate loaded_ mean the intermediate left after the reaction and the intermediate loaded into the reactor by weight, respectively; *m*_solid residue_ and *m*_catalyst_ represent the weight of solid collected after the reaction and the weight of catalyst introduced, respectively; *Y*_pi_ is the yield of product i by hydrodeoxygenation; *n*_pi_ and *C*_pi_ represent the moles of products i and carbon numbers of product i, respectively; and *n*_i_ and *C*_i_ indicate the moles of condensation intermediate loaded and carbon number of condensation intermediate, respectively.

The turnover frequency (TOF) of the Pd/C based catalysts was determined by using the following equation:

5 TOF (s^−1^) = converted FF-CP-FF in mmol/((exposed Pd atom) × (acid in mmol) × second)

## 3. Results and discussion

### 3.1. Catalyst characterization

**Fig. 1:**
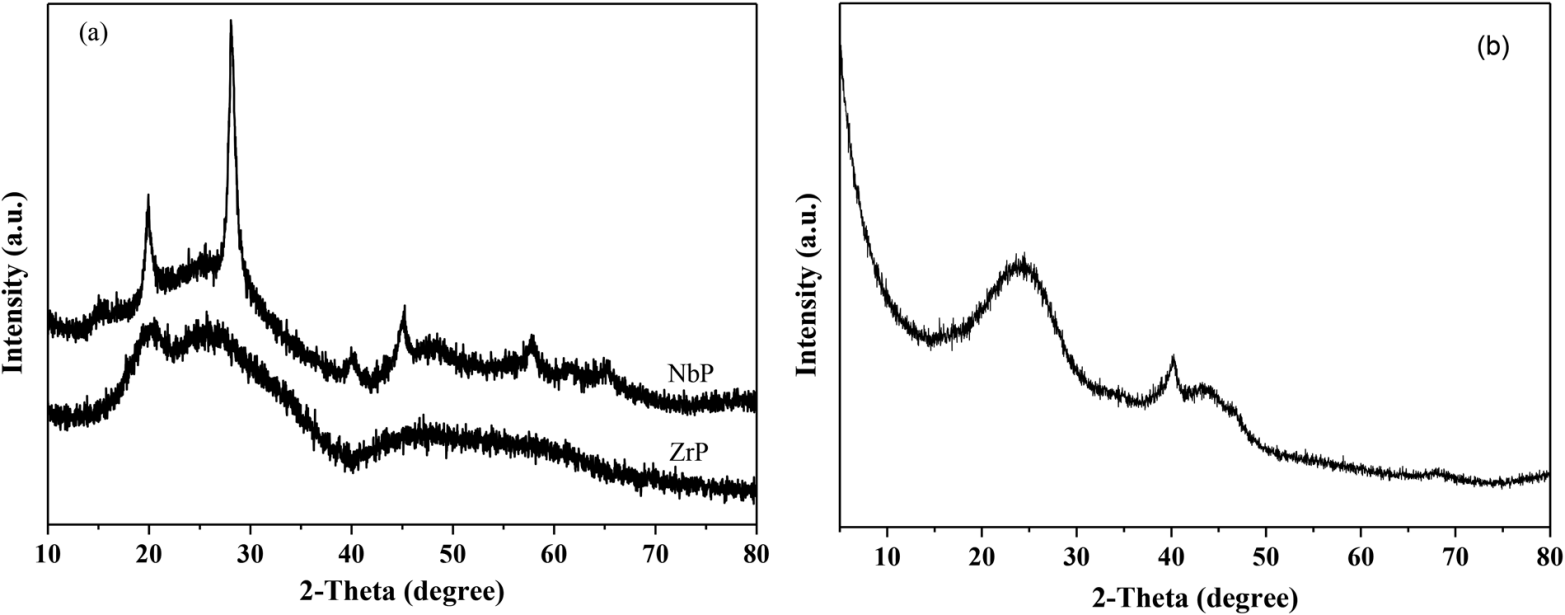
XRD patterns of fresh ZrP and NbP (a), and fresh Pd/C (b).

Fig. 1 shows the XRD patterns of freshly prepared NbP, ZrP and Pd/C catalysts. The obvious diffractions at 2*θ* = 19.8°, 28.0°, 40.2°, 44.9°, 57.8° and 65.1° indicate the crystalline nature of NbP, consistent with the previous report.^41^ On the other hand, the very weak and broadened diffractions observed in the range of 15–70° for ZrP, show that ZrP presents an amorphous nature. For Pd/C, peaks at 2*θ* = 40.1°, 43.9° and 68.3° were obtained but the intensities were weak, implying that the Pd particles are tiny and highly dispersed on the activated carbon surface.

**Fig. 2:**
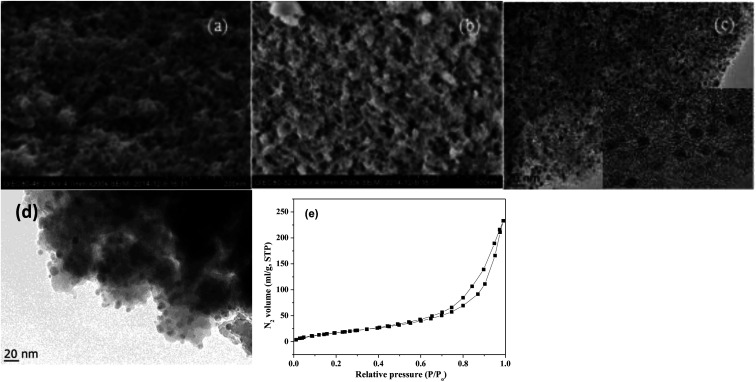
SEM images of fresh NbP (a) and ZrP (b). TEM images of Pd/C (c) and ZrP (d). N_2_ adsorption–desorption curve of ZrP (e). The inset in (c) shows the HRTEM image of Pd/C.

**Fig. 3:**
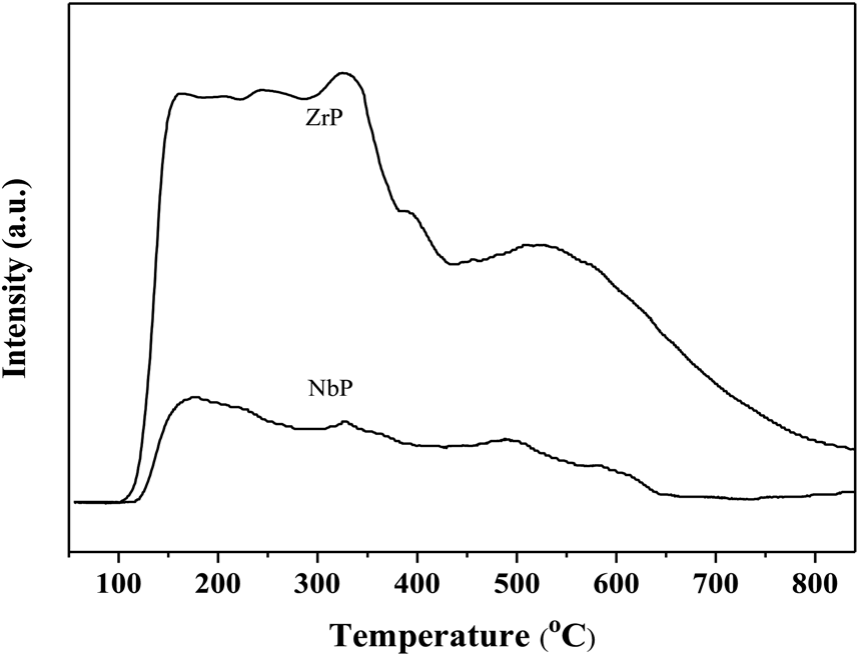
NH_3_-TPD profiles of fresh ZrP and NbP.

The SEM images of NbP and ZrP, the TEM images of Pd/C and ZrP, and the N_2_ adsorption–desorption curve of ZrP are shown in Fig. 2. Based on the SEM analysis, the particle size of the NbP was in the range of 10–20 nm, and that of ZrP was about 20 nm. The particle size of ZrP was consistent with the results obtained from TEM measurement. For Pd/C, the Pd particles were observed and their size was in the range of 2–3 nm, which is consistent with the result of XRD. As shown in Fig. 2e and Table 1, the surface area, pore capacity and diameter of ZrP were 112 m^2^ g^−1^, 0.25 mL and 6.8 nm, respectively, which are larger than those of NbP aside from the pore diameter. Both NbP and ZrP showed similar acidity, and both weak acidic sites (observed from NH_3_ desorption at temperatures below 350 °C) and strong acidic sites (observed from NH_3_ desorption at temperatures higher than 450 °C) were simultaneously presented on the two catalysts' surfaces (Fig. 3). As compared with NbP (0.58 mmol g^−1^), ZrP presented a much higher acid density of 1.55 mmol g^−1^ (Table 1). The Pd/C showed a large surface area of more than 1000 m^2^ g^−1^ with significant numbers of mesopores. Meanwhile, the Pd dispersion was as high as 0.49 according to the H_2_-chemisorption analysis. After hydrothermal treatment and recalcination, the surface area and acidic sites of the used ZrP were reduced, but more than 80% of the original values were still retained while the pore volume and diameter changed a little. The surface area of the used Pd/C was 90% of that of the fresh catalyst. The Pd dispersion decreased to 0.36, although high dispersion still remained during the hydrothermal treatment.

**Table 1:** Textural properties, acidic densities and metal dispersion of solid acids and Pd/C

Entry	Catalyst	Surface area^a^ (m^2^ g^−1^)	Pore volume^b^ (cm^3^ g^−1^)	Average pore diameter^b^ (nm)	Acidic density^c^ (mmol g^−1^)	*D* d
1	ZrP	112.7	0.25	6.8	1.55	—
2	Spent ZrP^e^	93.9	0.27	5.6	1.31	—
3	NbP	78.1	0.20	7.9	0.58	—
4	Pd/C	1025.9	0.28	3.8	—	0.49
5	Spent Pd/C^e^	932.5	0.25	4.3	—	0.36

a The surface areas were estimated by the BET method using N_2_ at −196 °C.

b The pore volume and average pore diameter were determined by using the desorption branch and BJH method.

c The acidic densities were calculated by NH_3_-TPD measurements and a calibration loop of 250 μL.

d The dispersion of Pd was determined by H_2_-chemisorption.

e The spent ZrP was treated at 300 °C for 3 h, dried and recalcinated at 400 °C for 4 h. The spent Pd/C was treated at 300 °C for 3 h, dried and recalcinated at 300 °C for 3 h under H_2_ flow.

**Fig. 4:**
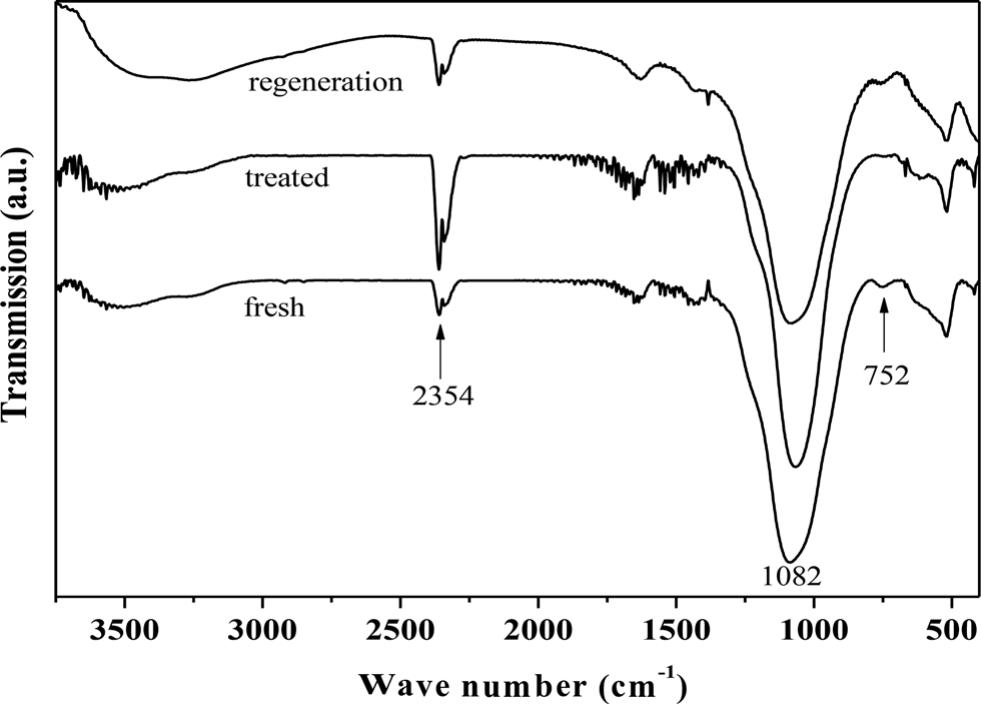
FT-IR spectra of fresh, hydrothermally treated, and regenerated ZrP.

Fig. 4 presents the FT-IR spectra of fresh, hydrothermally treated, and regenerated ZrP. The three samples presented the stretching vibrations of P–OH (the Bronsted acidic centers, 2354 cm^−1^), and tetrahedral P–O (1082 cm^−1^), and the bending vibration of P–O–P (752 cm^−1^), similar to the previous reports.^42–45^ After hydrothermal treatment, the peak intensity corresponding to P–O–P significantly reduced while that corresponding to P–OH increased, indicating that ZrP hydrolysis occurred during the treatment process. After recalcination, the P–O–P was regenerated at the expense of P–OH, based on the corresponding intensity changes. This means that the hydrolyzed P–OH could be re-condensed to recover the original structure. It was noted that the peak associated with the Zr–O–P framework was largely unchanged for the three samples, showing the hydrothermal stability of the prepared ZrP.

### 3.2. Condensation of furfural species with cyclic ketones

Aldol condensations of biomass-derived aldehydes (FF and HMF) with cyclic ketones (CP and CH) with mole ratios of 2 : 1 were used to synthesize C_10_–C_18_ precursors for downstream long-chained cycloalkane production. The condensation mechanism using FF and CP as an example is shown in Scheme S1.† The production of FF-CP-FF originates from consecutive C–C bond formation *via* a classical carbanion mechanism followed by dehydration over a base catalyst.

We began our study by choosing the aldol condensation of FF and CP as the model reaction to investigate. Various alkali catalysts (LiOH, NaOH, KOH, Ba(OH)_2_, Mg(OH)_2_, Na_2_CO_3_, Na_2_CO_3_) were used to catalyze this reaction at 30 °C (Table 2). The aldol condensation of FF with CP could not take place without any catalyst. However, when only a 5% molar equivalent of NaOH (relative to CP) was added into the mixture of FF and CP (at a FF : CP mole ratio of 2 : 1), the reaction took place quickly, producing a 68% yield of solid product in 40 min. After increasing the NaOH dosage to 10%, the conversion of FF and CP reached 100% (based on GC measurements; data not shown), and was accompanied by a high FF-CP-FF yield of 94%. Further increasing the NaOH to 20% slightly increased the yield of isolated condensation product FF-CP-FF to 96%. Other strong bases such as LiOH, KOH and Ba(OH)_2_ also showed excellent performance resulting in target product yields of 90% and above. However, no condensation product was observed when using the weak alkalis Mg(OH)_2_, Na_2_CO_3_ and K_2_CO_3_ as the catalysts even after 2 h, showing that such condensation preferentially takes place over a strong base. Because the strong base NaOH showed an excellent performance in the aldol condensation of FF with CP, we further employed NaOH for aldol condensations of FF with CH, and HMF with CP/CH. No obvious difference was observed when CH was used to replace CP as the starting material, which is in accordance with results from a previous study.^46^ Condensation products (FF-CP-FF and FF-CH-FF) were obtained in yields of over 90% from the condensation of FF with both CH and CP. When HMF was used as the starting material instead of FF, isolated condensation products (HMF-CP-HMF and HMF-CH-HMF) were obtained in over 95% yield. The condensation intermediates were yellowish for FF-CP-FF, FF-CH-FF and HMF-CH-HMF, and red for HMF-CP-HMF, as seen in the photographs of the isolated solid intermediates (Fig. S1†). The molecular structures of these intermediates were determined by ^13^C NMR measurement, which confirmed the synthesis of the desired products by aldol condensation without any impurities (Fig. S2–S4 and S6(a)†).

**Table 2:** Condensation of furfural species and cycle ketones over base catalysts^a^

Entry	Furfural species	Cyclic ketones	Catalyst dosage^b^ (mol%)	Time (min)	Product	Isolated yield^c^ (C-mol%)
1	FF	CP	—	40	FF-CP-FF	0
2	FF	CP	NaOH, 5	40	FF-CP-FF	68
3	FF	CP	NaOH, 10	40	FF-CP-FF	94
4	FF	CP	NaOH, 20	40	FF-CP-FF	96
5	FF	CP	KOH, 10	40	FF-CP-FF	93
6	FF	CP	LiOH, 10	40	FF-CP-FF	87
7	FF	CP	Ba(OH)_2_, 10	40	FF-CP-FF	92
8	FF	CP	Mg(OH)_2_, 10	120	FF-CP-FF	0
9	FF	CP	Na_2_CO_3_, 10	120	FF-CP-FF	0
10	FF	CP	K_2_CO_3_, 10	120	FF-CP-FF	0
11	FF	CH	NaOH, 10	40	FF-CH-FF	92
12	HMF	CP	NaOH, 10	40	HMF-CP-HMF	96
13	HMF	CH	NaOH, 10	40	HMF-CH-HMF	98

a All the reactions were conducted at 30 °C and the mole ratio of furfural compounds to cyclic ketone was 2 : 1.

b Relative to cyclic ketone.

c The isolated yield was calculated for the target product based on the cyclic ketone.

### 3.3. Long-chain cycloalkanes by hydrodeoxygenation of condensation intermediates

After obtaining the intermediates from the condensation of furfural species with cyclic ketones, we explored the hydrodeoxygenation of the condensation intermediates to cycloalkanes in an autoclave equipped with a mechanical stirrer.

Solvent is necessary for the hydrodeoxygenation process because the condensation products are all solid. The common oxy-organic solvents (methanol, ethanol and tetrahydrofuran) are not suitable because they could be digested under the hydrodeoxygenation conditions. Water is comparatively stable during intermediate hydrodeoxygenation, and is suitable as a green and economical solvent. Supported metal combined with solid acid is typically used as an effective catalyst for this process. For example, Huang *et al.* reported that carbon-supported noble metals (Pd/C and Pt/C) combined with solid acids (NbOPO_4_ and TaOPO_4_) are efficient in dehydration/hydrogenation of fuel precursors into liquid alkanes with water as the reaction medium.^47^

**Fig. 5:**
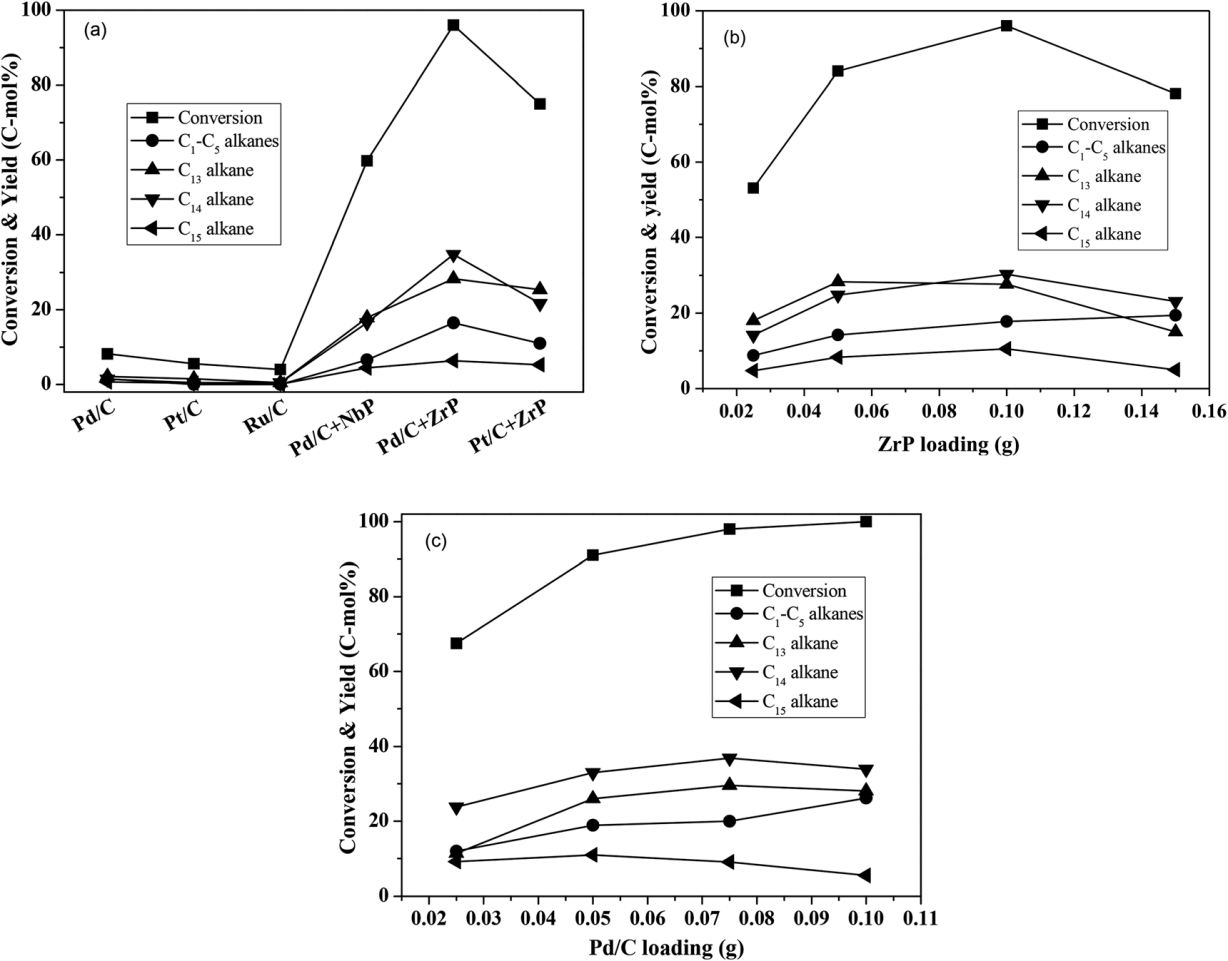
Hydrodeoxygenation of the FF-CP-FF intermediate to hydrocarbons over different solid acids and metals supported on activated carbon catalysts (a), with different ZrP loadings (b), and with different Pd/C loadings (c). Reaction conditions: 1.0 mmol FF-CP-FF, 15 mL H_2_O, 0.05 g metal supported on carbon, 0.10 g solid acid, 4 MPa H_2_ pressure, 300 °C, 3 h.

Using water as the solvent, we employed a one-pot hydrodeoxygenation of the FF-CP-FF intermediate into long-chained cycloalkanes. A low intermediate conversion of less than 10% as well as negligible cycloalkane production (with a summed yield below 5%) were obtained when using Pd/C, Pt/C or Ru/C alone as catalysts (Fig. 5a), suggesting that oxygen removal is a main barrier for alkane formation in the current reaction conditions. The addition of solid acid significantly increased the conversion and the cycloalkane yield. We calculated the average TOF of Pd/C combined with ZrP and NbP catalysts from the Pd dispersion, respective acidic sites and FF-CP-FF conversion at 3 h. The values of 0.47 s^−1^ and 0.87 s^−1^ were obtained for Pd/C plus ZrP, and Pd/C plus NbP, respectively; NbP has a much lower value for acidic density. Fixing Pd/C as the hydrogenation catalyst, 38.7% and 70.0% of cycloalkanes were obtained with the addition of NbP and ZrP, respectively, evidencing their obvious promotion effect in this process. The yield of cycloalkanes obtained with ZrP was much higher than that with NbP, because the superior acidic properties of ZrP could enhance dehydration and produce more cycloalkanes. As shown in Fig. S7,† the cycloalkanes with 13–15 carbon atoms were the main products apart from some minor oxygenated compounds at retention times of more than 15 min. Because the carbon chain of the FF-CP-FF intermediate contains 15 carbon atoms, the formation of C_13_ and C_14_ was ascribed to hydrocracking during the hydrodeoxygenation process. Among these products, the C_13_ and C_14_ cycloalkanes presented as the major products. 54% of C_13_–C_15_ cycloalkanes were produced when using Pt/C combined with ZrP as the catalyst. This indicates that Pd/C and ZrP are preferable for this hydrodeoxygenation. Additionally, gas products including C_1_–C_5_ light alkanes were observed; the existence of solid acid promotes the formation of such products.

ZrP and Pd/C loadings were further investigated and the results are shown in Fig. 5b–c. With increasing ZrP from 0.025 g to 0.1 g (fixing the Pd/C dosage at 0.05 g), the intermediate conversion and cycloalkane yield significantly increased from 53% to 96% and 47–76%, respectively, suggesting that the acid catalyst ZrP plays the critical role in oxygen removal. However, further increasing ZrP to 0.15 g led to a significant decrease in the cycloalkane yield, to 43%, as well as reduced conversion, which is perhaps caused by the intermediate coking and/or the produced cycloalkane hydrocracking to short-chain alkanes. In addition to the target products, the light alkanes (mostly CH_4_, C_2_H_6_, C_3_H_8_ and trace butane, pentane) increased with increasing ZrP and reached the highest proportion of 19% at 0.15 g ZrP. When the ZrP amount was fixed at 0.1 g, the cycloalkane yield increased with increasing amounts of Pd/C and the highest yield of 74% was obtained at 0.075 g Pd/C. When Pd/C was further increased to 0.1 g, the target products decreased but to a lesser degree than occurred with excess ZrP. This result shows that hydrocracking more likely occurred over solid acid. Based on the conversion efficiency and detected products by GC analysis, more than 90% of the carbon balance was obtained under the optimized conditions, showing that coking was suppressed during the process. At the same time, a decreased carbon balance of about 80% was observed in the case of excess ZrP or at a lower ratio of Pd/C to ZrP indicating that coking takes places with excessive solid acid. It is noted that the highest yield of 76% of cycloalkanes is comparable to that obtained in previous reports.^18–20^

**Fig. 6:**
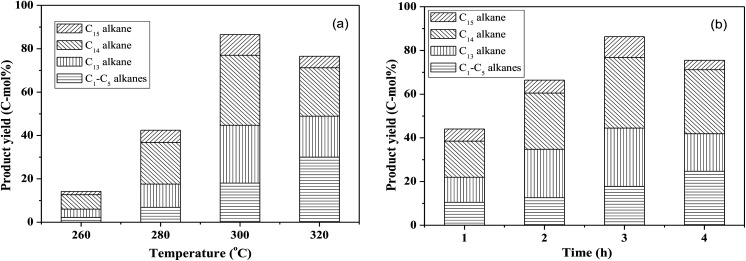
The influence of reaction temperature (a) and time (b) on the hydrodeoxygenation of the FF-CP-FF intermediate over ZrP and Pd/C. Reaction conditions: 1.0 mmol FF-CP-FF, 15 mL H_2_O, 0.05 g Pd/C, 0.10 g ZrP, 4 MPa H_2_ pressure.

**Fig. 7:**
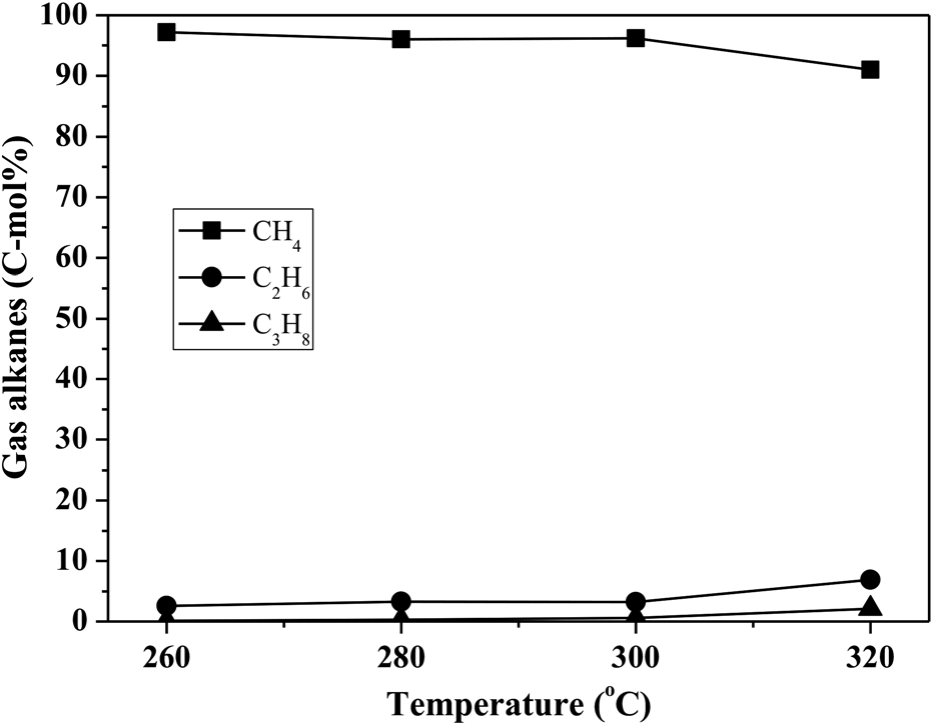
The influence of reaction temperature on gas product distribution in FF-CP-FF hydrodeoxygenation. The reaction conditions are as stated for Fig. 6(a).

By using the combined catalyst (0.05 g Pd/C and 0.1 g ZrP), we studied the influences of reaction temperature and time on cycloalkane production from the FF-CP-FF intermediate (Fig. 6). At a low temperature of 260 °C, a total cycloalkane yield of only 13% was obtained, but this greatly increased to 36% and 70% when increasing the temperature to 280 °C and 300 °C, respectively. However, further increasing the reaction temperature to 320 °C, decreased the cycloalkane yield to 49%. Additionally, the proportion of light alkanes increased with increasing temperature, reaching the highest value of 29% at 320 °C due to the enhanced hydrocracking at higher temperatures. Among these light alkanes, CH_4_ was selectively produced (>85% of the gaseous products) no matter what temperature was used (Fig. 7). The impact of reaction time was also investigated at 300 °C. At the short time of 1 h, the yield of C_13_–C_15_ cycloalkanes was 34%. The cycloalkane yield increased to 56% and 70% when prolonging the reaction time to 2 h and 3 h, respectively. However, further increasing the time led to a decreased cycloalkane yield. Similar to the effect of temperature, the lengthened time induced hydrocracking during the hydrodeoxygenation process and produced more light alkanes, resulting in reduced target products.

Hydrodeoxygenation of FF-CP-FF to produce cycloalkanes involves saturation of C=O/C=C bonds followed by oxygen removal. To ascertain this process, FF-CP-FF was hydrogenated into water-soluble oxygenated chemicals at the low temperature of 150 °C when using the single Pd/C catalyst (Fig. S5†) and the structures of the products were analyzed by the ^13^C NMR technique (Fig. S6†). The peaks at around 200 ppm, corresponding to C=O bonds, had not disappeared, suggesting that the C=O bonds in the FF-CP-FF were not efficiently hydrogenated into C–O bonds. The peaks in the range of 100 ppm to 170 ppm, which are associated with C=C bonds, were greatly decreased, indicating the hydrogenation of C=C bonds to C–C bonds. For the products obtained at the higher temperature of 250 °C, the peaks higher than 100 ppm all disappeared, confirming the saturation of these C=C and C=O bonds into C–O and C–C bonds, respectively. The quantified analysis shown in Table 3 further demonstrated the sequential C=C and C=O bond hydrogenation. At the reaction temperature of 300 °C, most of the oxygen was removed by cracking the C–O bonds, with only 3.5% of C–O bonds remaining in the final products. Introduction of solid acid promotes the dehydration of hydrogenation-derived C–OH, to produce the target products with high yields.

**Table 3:** Functional groups in FF-CP-FF and in samples hydrogenated over Pd/C at different temperatures, as determined by ^13^C NMR analysis

	C=O (%)	C=C (%)	C–O (%)	C–C (%)
FF-CP-FF	6.8	80.1	0	13.1
Hydrogenation at 150 °C	1.9	0.8	26.3	72.2
Hydrogenation at 250 °C	0	0	17.2	82.6
Hydrogenation at 300 °C	0	0	3.5	96.6

**Fig. 8:**
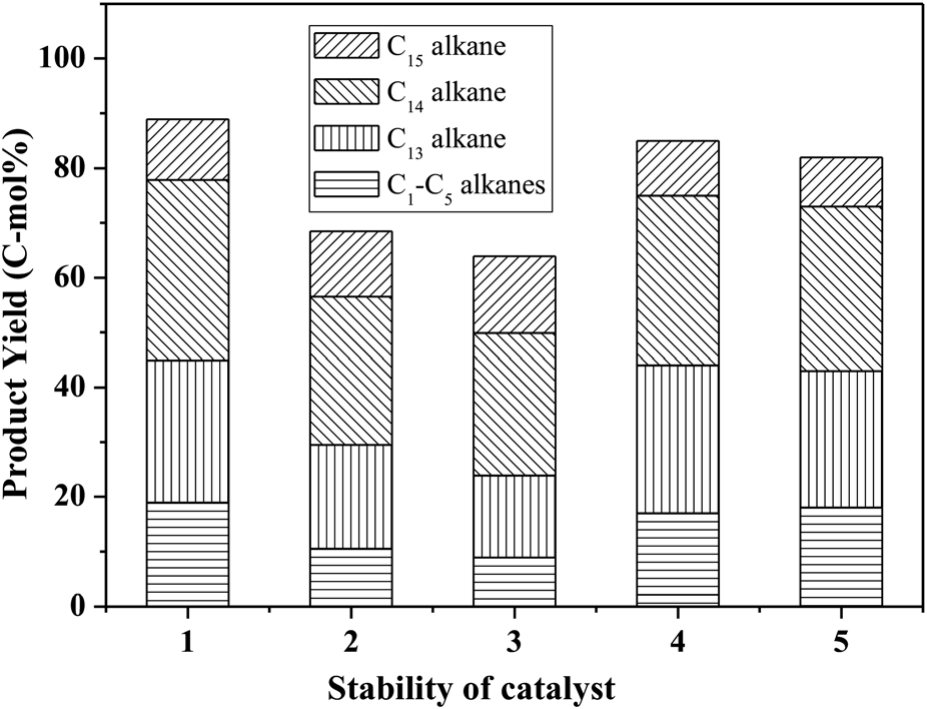
Catalytic stability of ZrP and Pd/C in the hydrodeoxygenation of FF-CP-FF to cyclic alkanes. Reaction conditions: 1.0 mmol FF-CP-FF, 15 mL H_2_O, 0.05 g Pd/C, 0.10 g ZrP, 4 MPa H_2_ pressure, 300 °C, 3 h. Bars 1–3 show successive cycles, bar 4 represents the regenerated ZrP combined with fresh Pd/C and bar 5 represents the regenerated Pd/C combined with fresh ZrP.

**Fig. 9:**
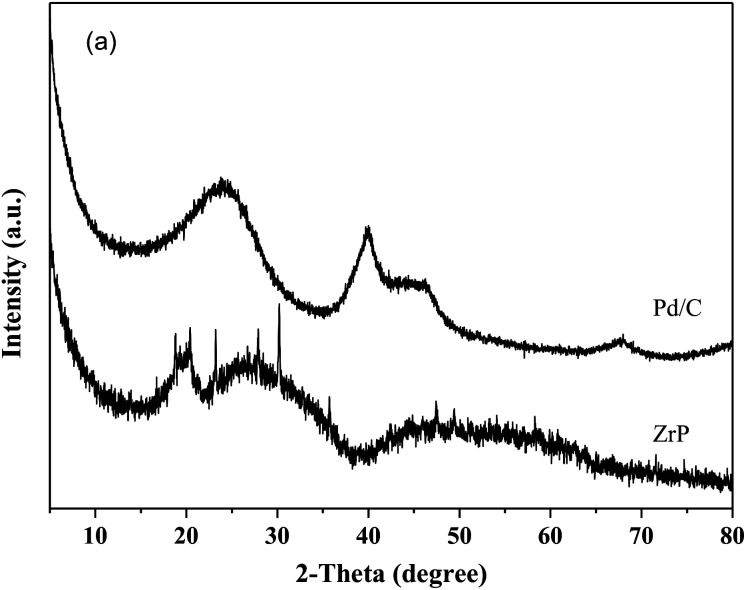
XRD patterns of hydrothermally treated ZrP and Pd/C followed by recalcination.

Fig. 8 shows the catalytic stability and regeneration performance of Pd/C combined with ZrP. The cyclic alkanes decreased from the initial 70% to a stable yield of 57% while simultaneously the light alkanes reduced from the initial 19% to below 10% during cycling. This decrease is due to a reduction in the acidic sites caused by P leaching in this hydrothermal hydrodeoxygenation according to our previous report.^48^ Considering that mixed Pd/C and ZrP are hardly separable from each other, we simulated the catalyst regeneration by hydrothermal treatment of the individual Pd/C and ZrP followed by recalcination according to the optimum reaction conditions. Interestingly, no matter which catalyst was regenerated, the activity of the combined catalyst could be recovered to the original level, demonstrating the excellent regeneration performance. After regeneration, the ZrP showed a similar amorphous nature to the fresh catalyst (Fig. 9). The weak and broad diffractions of the regenerated Pd/C show that Pd particles were still highly dispersed on the carbon surface although their intensities were slightly higher than those in the fresh catalyst. The good retention of the physicochemical properties for both regenerated catalysts is the main reason for their excellent performance.

**Fig. 10:**
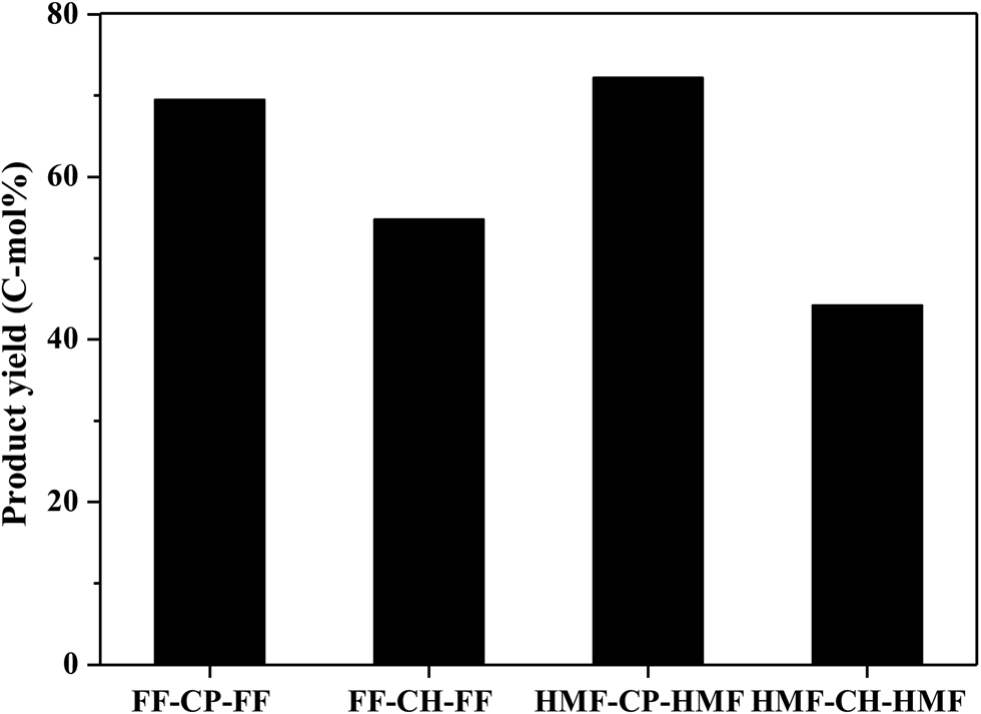
C_13_–C_18_ cycloalkane production from various condensation intermediates. Reaction conditions: 1.0 mmol condensation intermediates, 15 mL H_2_O, 0.05 g Pd/C, 0.1 g ZrP, 4 MPa H_2_ pressure, 300 °C, 3 h.

Hydrodeoxygenation of other aldol condensation intermediates (FF-CH-FF, HMF-CP-HMF, HMF-CH-HMF) were also investigated under the optimized reaction conditions by using Pd/C combined with ZrP as the catalyst. Cycloalkanes with carbon chain lengths of between 13 and 18 were obtained. In the cases of FF-CH-FF and HMF-CP-HMF, 55% and 72% yields, respectively, of cycloalkanes were obtained. When using HMF-CH-HMF as the feedstock, a 43% yield of cycloalkanes was obtained. This indicates that the developed catalyst of Pd/C combined with ZrP is generally efficient for the production of jet fuel and/or diesel hydrocarbons by hydrodeoxygenation of these condensation intermediates (Fig. 10).

## 4. Conclusions

Long-chained cycloalkanes with carbon chain lengths of 13–18 were obtained from furfural species (FF and HMF) and cyclic ketones (CP and CH), all of which could be obtained from the renewable lignocellulosic biomass. The aldol condensation of the furfural species and cyclic ketones was conducted at 30 °C with a strong basic catalyst, to obtain over 90% yields of the desired condensation intermediates. The long-chained cycloalkanes with carbon chain lengths of 13–18 were produced *via* hydrodeoxygenation of the condensation intermediates at temperatures of 280–320 °C and times of 1–4 h by using a catalyst of Pd/C combined with the solid acid ZrP. Under the optimized conditions, the highest yield of 76% of target products could be obtained. The combined catalyst showed reduced yields of cycloalkanes after the second and third cycles, but its performance could be largely recovered to its original level by hydrothermal treatment followed by recalcination of Pd/C and ZrP.

## Conflicts of interest

There are no conflicts to declare.

## Supplementary Material

RA-008-C8RA01723A-s001
