# Supplementary material for: Production of renewable long-chained cycloalkanes from biomass-derived furfurals and cyclic ketones
Source: RSC Adv. 2018 Apr 12;8(25):13686–96. doi: 10.1039/c8ra01723a (PMC9079845; doi:10.1039/c8ra01723a)
Supplement: RA-008-C8RA01723A-s001 [file RA-008-C8RA01723A-s001.pdf]

Electronic Supplementary Material (ESI) for RSC Advances.
This journal is © The Royal Society of Chemistry 2018

Supplementary material

Production of renewable long-chained cycloalkanes from biomass-derived furfurals and cyclic ketones

Qiying Liu [a,*], Caihong Zhang[a,b], Ning Shi [a,b], Xinghua Zhang[a,*], Chenguang Wang [a], Longlong Ma [a]

[a] *CAS Key Laboratory of Renewable Energy, Guangzhou Institute of Energy Conversion, Chinese Academy of Sciences, Guangzhou, 510650, P. R. China and*

[b] *University of Chinese Academy of Sciences, Beijing, 100049, P. R. China*

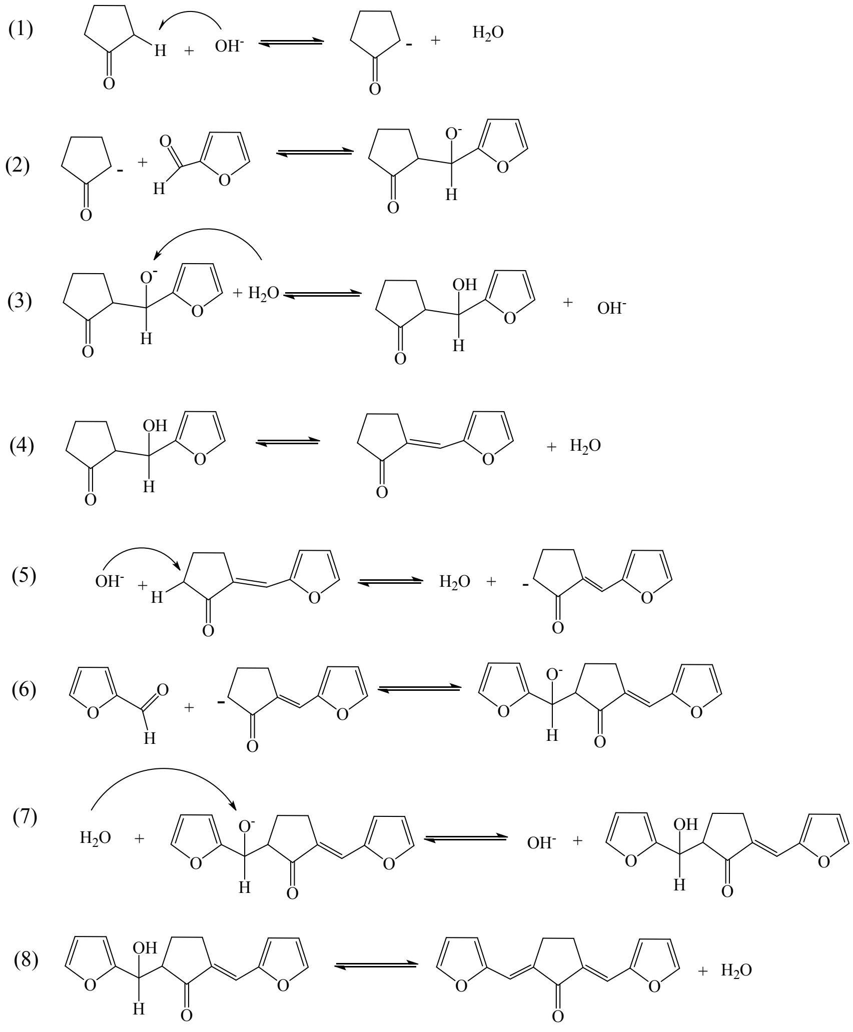

Scheme S1 Formation mechanism of FF-CP-FF by aldol condensation of CP and FF.

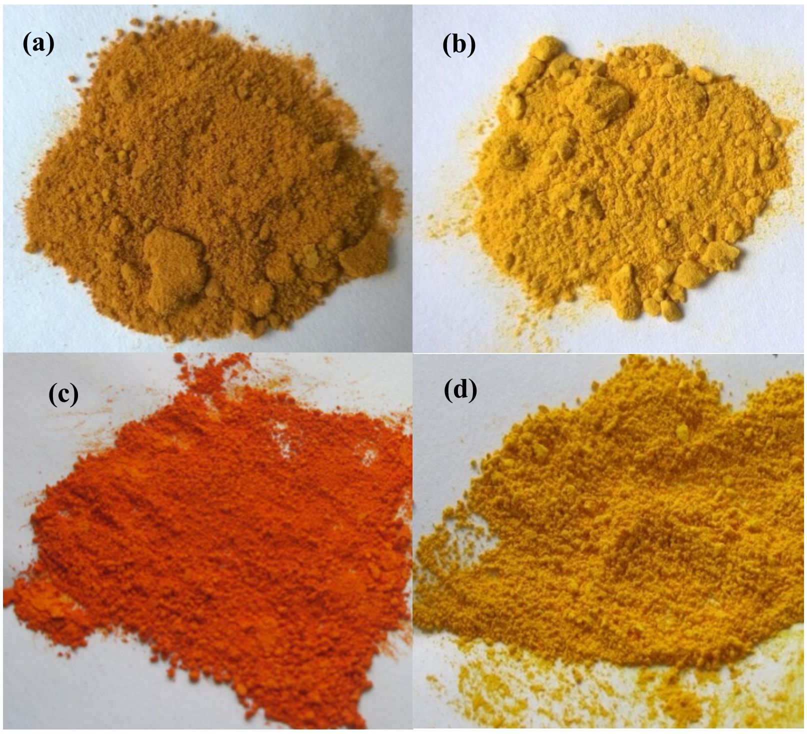
(a)
(b)
(c)
(d)

Fig. S1 Photographs of the condensation intermediates: (a) FF-CP-FF; (b) FF-CH-FF; (c) HMF-CP-HMF; (d) HMF-CH-HMF.

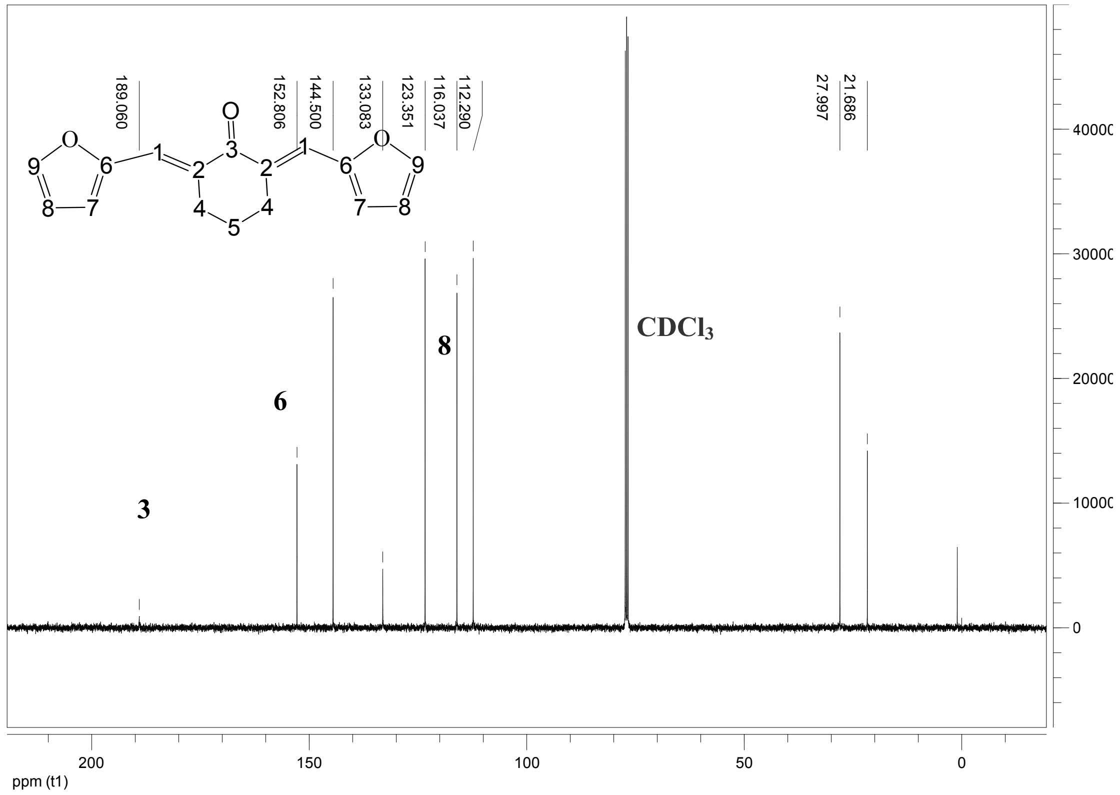

Fig. S2 The ^{13}C NMR spectrum of FF-CH-FF.

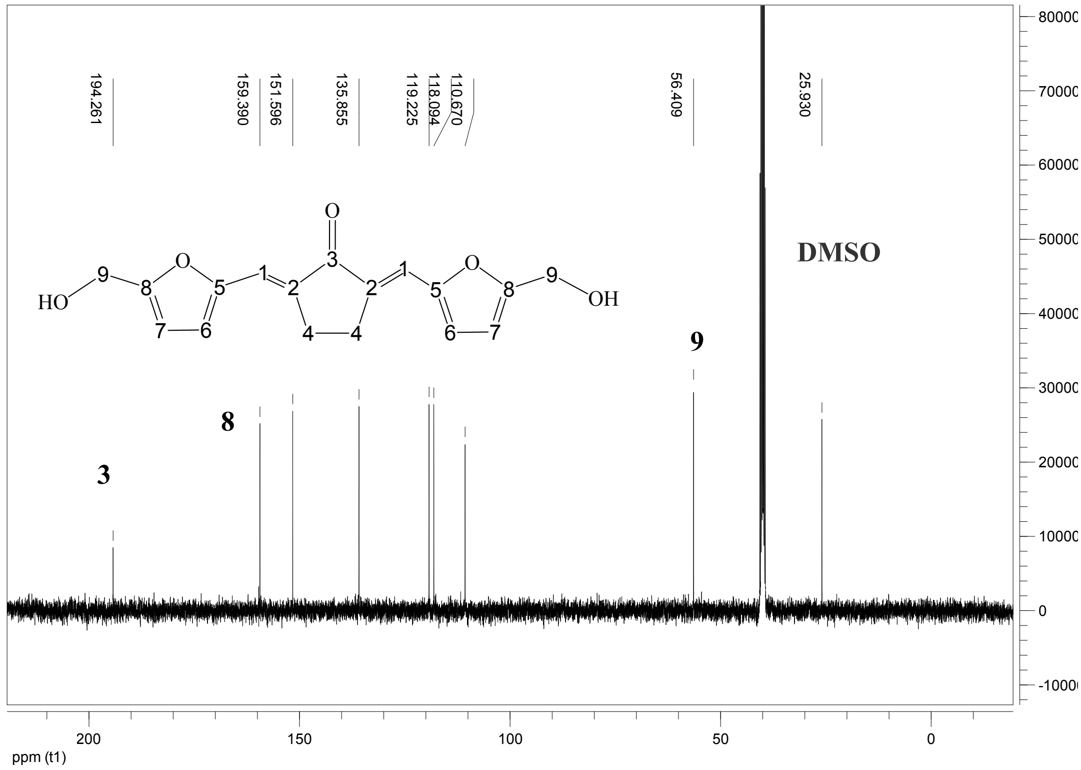

Fig. S3 The ^{13}C NMR spectrum of HMF-CP-HMF.

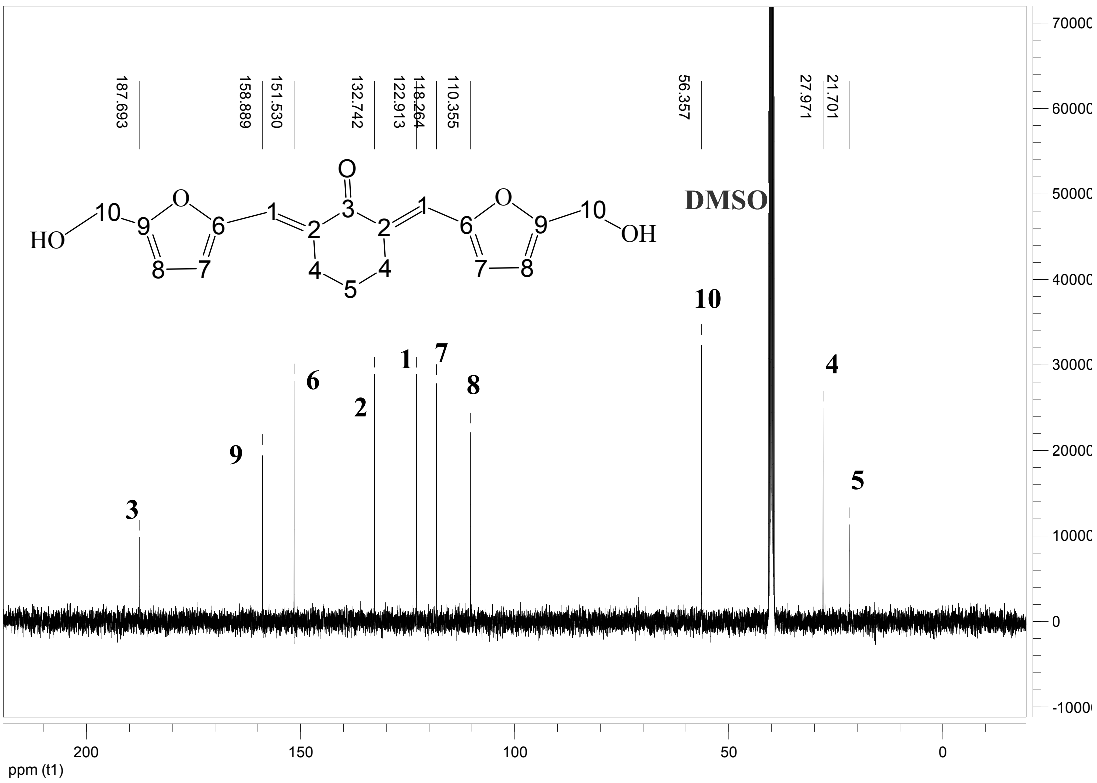

Fig. S4 The ^{13}C NMR spectrum of HMF-CH-HMF.

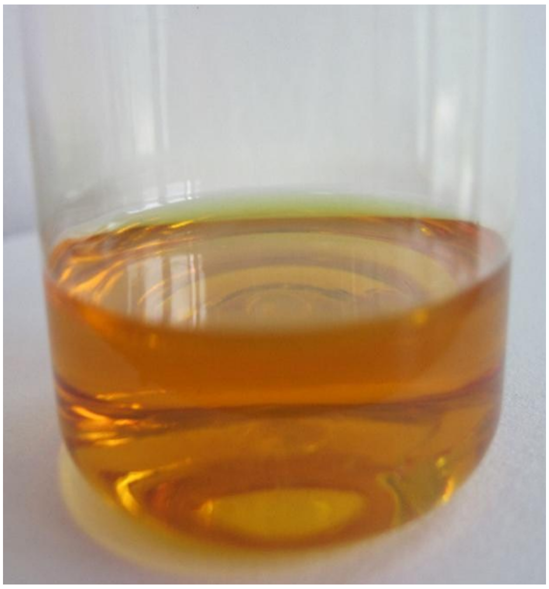

Fig. S5 Photograph of intermediate produced by FF-CP-FF hydrogenation at 150 ^{o}C over Pd/C. Reaction condition: 1 mmol FF-CP-FF, 15 mL H_2O, 0.05g Pd/C, 4 MPa H_2, 3 h.

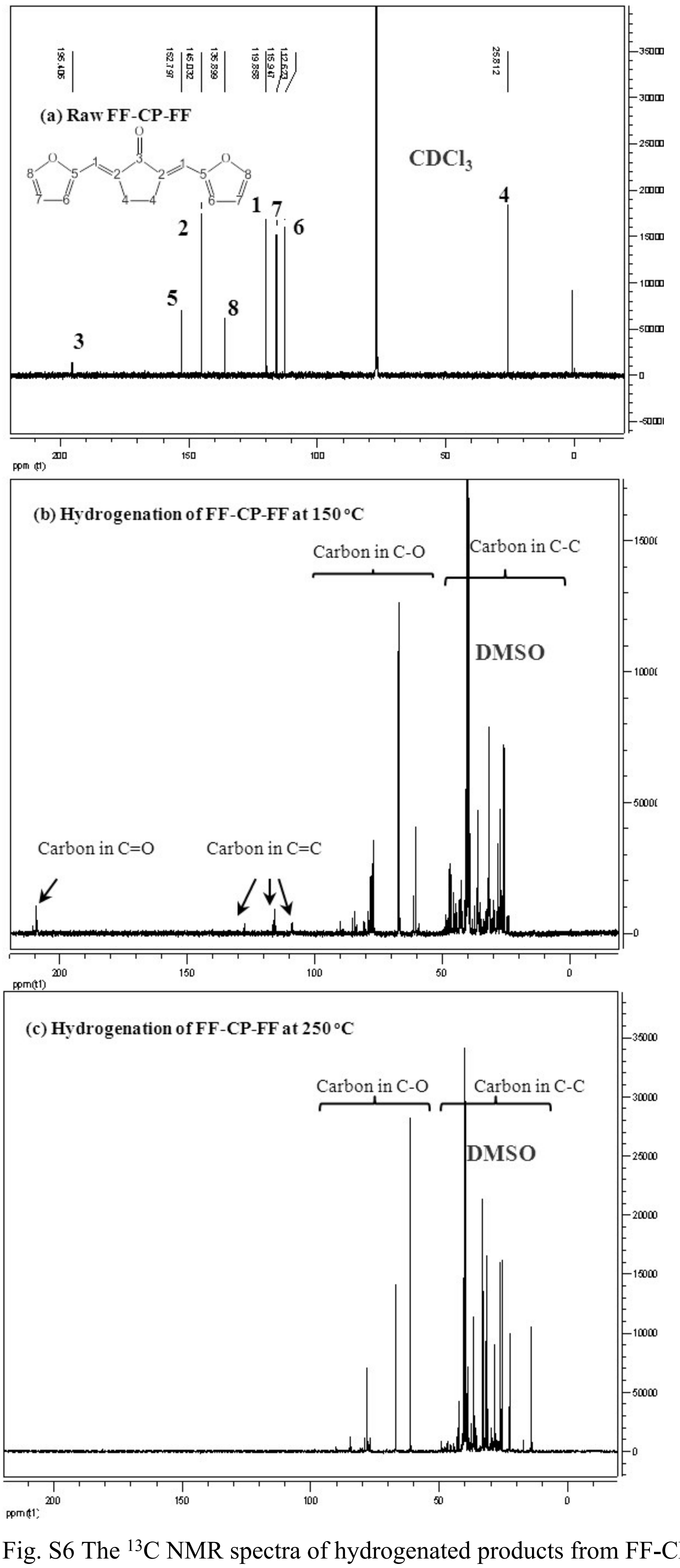

Fig. S6 The ^{13}C NMR spectra of hydrogenated products from FF-CP-FF at different temperatures. Reaction condition: 1 mmol FF-CP-FF, 15 mL H_2O, 0.05g Pd/C, 4 MPa H_2, 3 h.

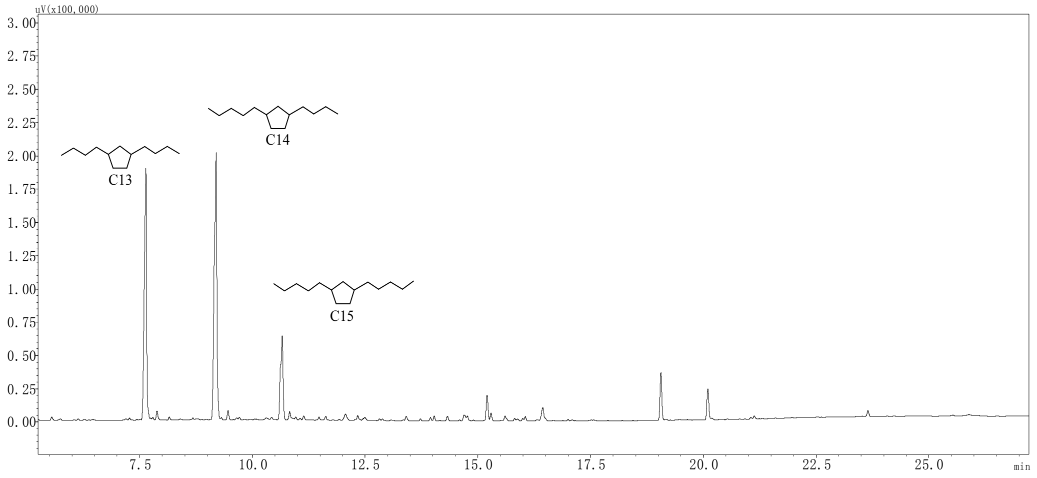

Fig. S7 Representative GC result from hydrodeoxygenation of FF-CP-FF. Reaction conditions: 1.0 mmol FF-CP-FF, 15 mL H_2O, 0.05 g Pd/C, 0.10 g ZrP, 300 ^oC, 3 h and 4 MPa H_2 pressure.
